# Realigning Health Systems Strategies and Approaches; What Should African Countries Do to Strengthen Health Systems for the Sustainable Development Goals?

**DOI:** 10.3389/fpubh.2020.00372

**Published:** 2020-08-07

**Authors:** Sunny Ibeneme, Moses Ongom, Nkiruka Ukor, Joseph Okeibunor

**Affiliations:** ^1^Federal Ministry of Health, Abuja, Nigeria; ^2^World Health Organization – Country Office, Abuja, Nigeria; ^3^World Health Organization – African Regional Office, Brazzaville, Congo

**Keywords:** Africa, action framework, health systems strengthening, universal health coverage, sustainable development goals

## Abstract

The African region is experiencing peculiar demographic, economic, social and environmental challenges that place pressures on the health systems. While the need to explore ways to address identified health systems challenges is far from easy, there are substantial evidence that having robust frameworks and metrics to direct efforts and priorities of countries could be rewarding. In view of persisting regional health systems' challenges the World Health Organization African regional office proposed the adoption of a comprehensive health system strengthening action framework that provides an opportunity to translate global health policy into operational strategies for Africa's health sector policies, strategies and operations. The adoption of the action framework could support the realization of regional health objectives and priorities, and guide movement toward sustainable developments in countries.

## Introduction

It is almost two decades now since the United Nations Millennium Development Goals (MDGs), which was set to be achieved by 2015 were signed and adopted among 189-member countries. These goals were ambitious. Though countries in the African region outpaced global trends, a lot were yet to be desired (1). Recent report from the Atlas of African health statistics documented improvements among several health indicators across this period. Life expectancy at birth rose from 50 years to 60 years between 1990 and 2015. Adult mortality rate/100,000 decreased from 400,000 to 200,000, and maternal mortality rate/100,000 dropped from 1000,000 to about 600,000 (2). While there are substantial improvements on health MDGs among African economies, agreed global averages and benchmarks were not achieved in the Region (3, 4).

Growing empirical evidence suggests that despite advancements in the availability of many priority health interventions, and an increased funding for health, progress toward agreed MDGs remain slow and retrogressive– suggesting weak and fragmented health systems, and operational failures among stakeholders as key barriers to achieving the MDGs (3, 4). Health systems investments are often not well-linked to expected health outcomes, which lead to a divide between service interventions and overall health systems focus. Most health investments are vertical and program-specific that undermine wider system effects (2, 5). In addition, most identified priorities are partner-driven with limited policy buy-in. Health systems and services provisions are not aligned to allow effective policy response to system challenges (5). Service delivery are largely disease-specific programs that focus on one or two health systems functions in isolation with no systematic evidence for positive and negative effects, necessitating the need for a holistic approach to program coordination using robust frameworks (2, 6).

The objective of this article is to contribute to the development of a better and more widely shared understanding of approaches that strengthens African health systems. This paper highlights peculiar challenges prevalent among African health systems, including policy responses to identified challenges using the World Health Organization Regional Office for Africa's (WHOAFRO) action framework. It explores approaches for operationalizing the WHOAFRO action framework, while highlighting policy recommendations to address identified challenges. The adoption of the framework provides an opportunity to translate global health policy into operational strategies for Africa's health sector policies, strategies and operations including Monitoring and Evaluation (M&E).

While effective health systems are prerequisite for advancing Universal Health Coverage (UHC), and health-related Sustainable Development Goals (SDGs), there is increasing systems verticalization among African health systems. There is little focus on system building and strengthening to drive the mandate of UHC and SDGs (4). Stakeholders too often adopt the reductionist perspective to system thinking that ignores important aspects with limited focus on system building (6). The quest to produce result for the SDGs have led stakeholders to focus on disease priorities, with the assumption that systems will be strengthened more when specific interventions are implemented for prioritized diseases. However, there is empirical evidence that if health systems holistically lack capabilities in health workforce, health financing, health information, health governance and service delivery, such systems may not be able to respond effectively to such opportunities (3). Besides, over-concentrating resources for specific programs may further compromise an already weak health system (6). Thus, there is need to strengthen health systems that integrates the different building blocks toward effective health service delivery and UHC. Health systems components should function cohesively to enhance universal coverage. Operational strategies and interventions should be designed to synergistically build robust, resilient and responsive health systems for optimal health outcomes.

As investments in health continue to increase over the years, and funders support for wider initiatives for health systems strengthening also increasing; there is need to know not only what works, but what works for whom and under what circumstances? If we accept that no intervention is simple, and that every intervention has positive and negative effects across systems, then it is important that we understand the full range of those effects to amplify possible synergies and mitigate impediments (7). We must understand the system to strengthen it and be able to design better interventions and strategies with system-wide effects.

## Evolving Peculiar Challenges Among African Systems: Increasing Complexity of Contexts

According to reports from the United Nations Development Program (UNDP), Africa's population is expected to reach 2.3 billion by 2050, with an estimated 830 million young people (8). If current trend persists, one in every four individuals on earth will be African by 2100 (9). With little economic diversification and weak education system, the chances that the teeming African youths could find productive employment and have a sustainable livelihood remains slim (5). This trend has further placed pressures on the burgeoning global population which is on the increase by about 75–80 million persons per year and is estimated to reach 10 billion before the end of the century (4).

In an era of slow economic developments among most African countries, there is need to take these demographics seriously and efforts made to actively re-orient high-fertility households to adopt voluntary fertility reductions for the benefit of the family, the environment and the economy. Recent study shows that one half of the African population live below poverty line (10). The per capita Gross Domestic Product (GDP) have declined over 11 percent since 1974. One in ten poor people lived in Africa in 1970, compared to 2000 where the ratio was one in two (10). These statistics continue to deteriorate, and are further worsened by persisting political instability, military dictatorship and institutionalized corruption among most African economies (11). The chaos in governance has perpetuated the continued change of stewards among political leadership, precipitating inconsistencies in system governance (12). These have led to the serial toppling of governments in the recent turbulent years, with loss of synergy between scientific and political groups, precipitating poor outcomes (11).

Beyond these socio-economic threats, the African region has also witnessed increase access to information, technologies and human rights along with slow economic growth and commodity-based economies. Increased penetration of new technologies and innovations comes with opportunities and threats in an era of increasing urbanization and globalization (4). There is increased scramble for water, food, jobs, and arable land. The youths find themselves aggrieved. They have been born in an era of technological advancements, which rather enhance their access to good jobs and opportunities threatens it (4). The youths are born in a region undergoing dangerous environmental and epidemiologic transitions and other serious climatic ills. Human activities have continued to push planetary boundaries past dangerous thresholds with devastating consequences for life and well-being (4). There is need to understand the changing epidemiology of diseases and recurring outbreaks of emerging and re-emerging diseases in all sustainable development dialogues.

These pressures are both local and international. They impact simultaneously on several aspects of the earth systems: water, nitrogen and carbon cycles. Mankind thus faces effects of climate change resulting from environmental pollution, acidification of water bodies, and deforestation to say the least. Various meteorological events and related environmental distress syndrome have led to the emergence of opportunistic pests and pathogens across a wide range of taxonomic animals and plants perpetuating the occurrence of emerging and re-emerging infectious diseases even in areas where they have been hitherto eliminated (13, 14). Prime examples are the Ebola epidemic of 2014 (15), Lassa fever outbreak of 2018 (16), and Corona virus pandemic of 2019 (17).

That notwithstanding, inequalities still persist among most African economies (2). Vulnerable and impoverished communities continue to endure extreme hardship and global response has not been ambitious enough (18, 19). There is stark discrimination against most indigenous black populations as the scramble for opportunities and good life intensifies. Only 36 percent of the 31.5 million people living with HIV in the developing parts of the world received antiretroviral therapy in 2013. This was attributed to poor supply and distribution mechanisms, low health sector investments and inefficient health systems. These breed low satisfaction with services, increase attrition rates and reduce the likelihood of use of services leading to poor health outcomes (18).

## Policy Response To Africa Health Systems' Challenges Using The Framework of Actions

**Figure 1:**
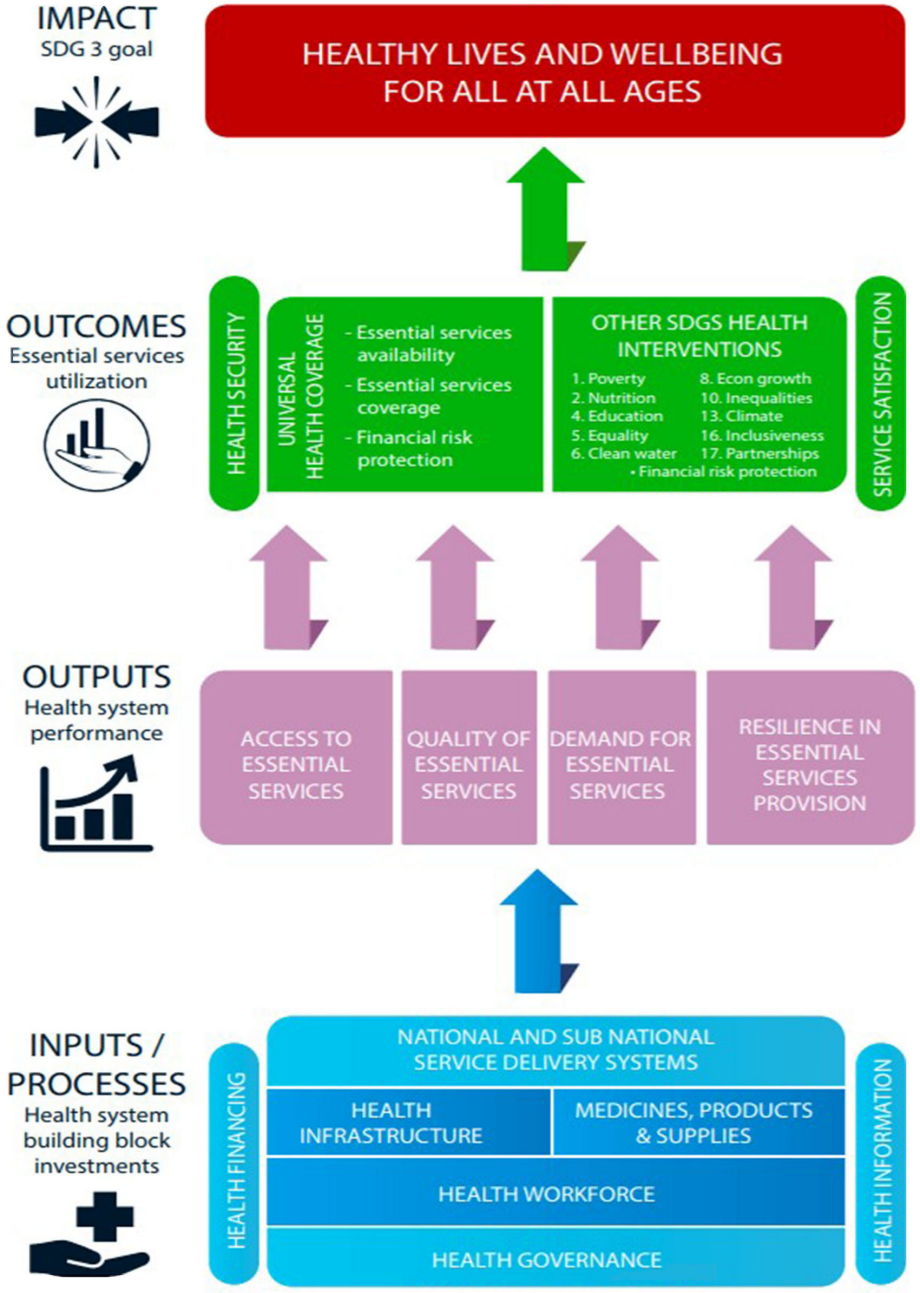
Framework of Actions for solving Africa's complex health systems challenges.

In view of the peculiar challenges prevalent among African economies, the need for an advanced change-producing approach is imperative. The WHOAFRO proposed the use of a comprehensive health systems strengthening framework (Figure 1) to solve peculiar regional challenges and align Health System Strengthening (HSS) to the needs of countries. The goal of the action framework is to guide African countries' efforts to re-align their health systems in a way that facilitate the realization of UHC and the SDGs. The framework uses system-wide approaches to integrate HSS elements for the goal of attaining sustainable development objectives of countries. It emphasizes important elements that countries should focus on and how countries can organize and channel efforts toward achieving prioritized elements. Evaluative tools and guidelines are developed in-line with planning process to facilitate the operationalization of the framework in countries. The action framework has a scoping element which maps forward threats, and an inbuilt flexibility with robust redesign processes that accounts for changes in present and future situations. It has clearly defined metrics for monitoring intermediate targets with timelines (2). Intermediate checks ensure proper alignment and timely feedback between formulated policies and prioritized health outcomes (20). Thus, in-line with WHOAFRO's commitment of working with countries to produce robust health systems suitable for individual country's contexts, needs and priorities, WHOAFRO established the institution of an annual Ministries of Health (MOH) Directors of Planning meetings, as well as routine scoping exercises on UHC with MOHs based on the framework (2).

The action framework has four domains defined around possible options of actions for countries. These are operational action points that target to assist countries in deciding priorities about the planning, implementation and M&E of their national health policies. Key domains of action include– Health systems input, output, outcome, and impact. Input domain defines points where health investments are made in the form of hardware and software that produce outputs and translates to defined outcomes and impacts. Related areas of action defined around inputs include–health workforce, health infrastructure, medical products and health technologies, health service delivery, health governance, health financing, and health information, research and innovation (2). Health output domain define standards by which health systems performance are measured. These are elements needed for effective service delivery of essential health interventions. Areas of performance defined around this domain include: Health system resilience, efficiency and equity of access, quality of care, and service demand (2). The third domain relates to population coverage outcomes with essential interventions including the vulnerable and marginalized groups. Prioritized health outcome areas include: Availability of essential services for life-course cohorts, coverage of essential services across related groups, health security, health financial risk protection, client satisfaction, and cross-sector coverage of essential health services with other SDGs (2). Lastly, the impact domain is related to the SDG 3– Healthy lives and well-being for all at all ages. This goal is expected to be achieved by all African nations by 2025. Elements contained in this thematic area include: Improvements in life expectancy, morbidity and mortality reductions, and risk factor reductions among individuals (2).

## Operationalizing The Action Framework: Crossing Health Systems Chasm

Translating global health policies into operations and results are big gaps among African health systems. African governments want to improve national health outputs and outcomes, but many lack capacity and knowledge on frameworks for designing comprehensive health systems strengthening interventions (2, 3). The adoption of the WHOAFRO framework provides an opportunity to translate robust health policies into operational strategies for Africa Member States to strengthen their health systems and achieve sustainable health outcomes. The action framework provides a comprehensive way for countries to integrate HSS needs and priorities for the realization of the SDGs. It requires governments of Member States to take ownership in identifying the needs of their country and implement the right interventions to build robust health systems that could contribute in achieving better health outcomes.

Murray and Frenk (21) documented the use of the WHO performance framework for assessing health systems performance, including its usefulness in identifying and addressing important HSS gaps (21, 22). This corroborated work done by Shakarish et al. (22) which documented the use of a comprehensive systems framework to design effective HSS interventions that have long-term positive impact on health services (22, 23). Thus, the WHOAFRO action framework has opportunities to improve regional health outcomes through an integrated approach to HSS (2). The framework guides and directs the development of countries' national health policies at various phases. At the planning phase, the framework helps define how actions are to be determined. It helps countries to identify long term objectives at different impact levels. At the implementation phase, the action framework guides countries on how to translate selected actions from inputs areas into meaningful activities. It provides standards for countries to evaluate implemented actions regarding their ability to achieve planned targets at various levels of outputs, outcomes and impacts. The M&E phase involves series of events including: Evaluation of input actions through routine activity reviews; evaluation of output targets as part of annual monitoring processes; evaluation of outcome targets through periodic program appraisal processes; and the evaluation of impact objectives through national M&E processes and assessments (2).

The application of the framework in framing national health policies is never linear in practice but has areas of overlap of actions and activities and is flexible depending on country contexts. Stakeholders in MOH are guided on how to operationalize the framework to navigate relevant processes when framing national health policies. The framework presents a holistic approach to strengthen health systems, that removes program-specific approaches of the MDG era. It empowers countries to ensure that health sector planning aligns with government development agenda and priorities; and fosters the alignment of national health policies with operational strategies that improves health systems at scale. Thus, the framework fosters linkages and interconnections that dissuades redundancy and silos approaches to health system thinking (24, 25). Conversely, the possible weaknesses and challenges of the framework will be its utility in Africa's widely variable contexts– Africa has contexts ranging from small island States (Seychelles) to large multi-State federations (Nigeria); High income countries (Equatorial Guinea) to fragile States (DRC). However, the framework has mechanisms adaptable to differing settings that allow Member State to tailor their approaches based on their contexts, capacity and priorities while allowing WHOAFRO provide the necessary technical support.

The question pertinent is whether African governments are living up to their responsibilities and commitments to strengthen national health systems, and whether that is having any impact or not. The metric for measuring progress is the rate of progress, and not the likelihood of achieving set targets (26). Thus, the important question for implementation is to understand where and why there is notable acceleration of progress in some countries (Rwanda, Ethiopia) and not others (Democratic Republic of Congo, Guinea Bissau). These are critical issues the action framework has opportunities to address through robust planning, implementation, and review mechanisms– Such as the establishment of health observatories, regular reviews, communities of practice, and capacity improvements support by WHOAFRO. Such mechanisms encourage governments to be accountable and committed as they are guided to prioritize health systems actions that ensure systems investments are linked to service outcomes using the framework (2).

## Moving Forward: Policy Recommendations To Strengthen African Health Systems For The SDGs

As the discussion for the SDGs gain momentum, it is important we begin to think how to put an increasingly fragile world on the path to sustainable development. Contrary to the strategy involved in the elimination of extreme poverty which involved advancements in essential technologies (medicine, high-yield seeds, electricity, diagnostics, and internet) from high- and middle-income countries to low income countries; realizing the SGDs will be different and could be phenomenally complex (5). The global community will need to identify critical pathways to organize human efforts and technologies to improve living standards and ecological imperatives which will drive the needed social change for both rich and poor countries. They are to pose goals and challenges for all nations– not what rich countries should do for poor countries but a global commitment to save this planet. This will entail adopting strategies that build on sustainable developments– green technologies, sound market incentives and clean energy systems (4). Global developmental agreements including “The Astana Declaration” and “The Paris Agreement” should be consolidated and evidence to guide implementation made available for global systems.

The global sustainable development agenda can benefit from the experiences of the MDGs in view of its successes and shortfalls. While the strengths are reinforced, strategies to address shortfalls should be put in place to enhance global awareness, political accountability, and improve metrics (4). Sustainable development mandate should be pursued through practical, actionable and specific measures with robust enforcement mechanisms. There is need to improve health systems at scale by redesigning imperatives using relevant models like the action framework. Strategies and interventions should be encompassing and assume micro-level perspectives. This will consolidate agreed priorities, and foster positive synergy among stakeholders– policymakers, the private sector and the scientific community among others. Politicians set priorities. Thus, such collaboration should be harnessed to re-align strategies that reposition health service delivery and strengthen health systems (27).

In addition, efforts should be consolidated to strengthen health systems policy research in the areas of practice, education and policymaking for effective systems (5). An excellent understanding of how to interact with policymakers, and what details would be required, in what form and with whom such interactions should be made will be needed. Factors that facilitate and impede translating research into policy and practice should be evaluated as to strengthen the relationship between researchers and policymakers. This helps to identify priorities that need active public participation, strong political focus and robust quantitative measurements (28, 29).

Sustainability requires responsibility, accountability and leadership. For the sustainable development agenda to thrive, global societies need to adequately invest in its success. It is the only viable hope for mankind, but it will never survive unless a small fraction of our consumption spending is saved for long-term investments for survival– not only for health but poverty reduction, climate change mitigation and conservation of biodiversity. All hands must be on deck. Private sector involvement should be encouraged and consolidated. Neither UHC nor the SDGs will be achieved without meaningful engagement of leadership from multinational companies–large and small. The private sector brings unique strengths and opportunities in the areas of innovative technologies and capacities to advance solutions that are central to success. Indisputably, most multinationals are lobbyist to proposals antagonistic to sustainable developments. Thus, business engagements should be done with caution but should be direct, forward and actively intensive (4).

Funding sustainable developments should be more focused, direct and realistic than was the case for the MDGs. Conceptually, SDGs funding largely rely on voluntary financing mechanisms (18). Free riding on foreign financial aid is the norm, but not an exception. Only a few countries abide by the promise to earmark 0.7% of their GDP for developmental assistance. Other related time-bound pledges on developmental assistance like those made at the G8 Gleneagles summit of 2005 were also not fulfilled by countries (4). Funding for the SDGs should be realistic and consolidated. Instead of countries to rely on aid voluntarism, they should agree to transparent and standard financing mechanisms including the International Monetary Fund and other related United Nations dues and levies, which relates to national incomes. This can complement private philanthropy and accrue for success (19).

Lastly, realizing the SDGs will involve unprecedented global mobilization of knowledge, responsibilities and resources. Stakeholders should work together to discuss crucial pathways to success in a manner that reflect democratic representation and technical expertise. The paths to meeting global sustainable developments can never be identified through a top-down approach, but through a highly motivated and energized network of problem-solving cohorts comprising the academia, governments, the private sector, Non-governmental Organizations, civil society, businesses, and importantly the youths who expectedly should be experts and leaders of a new profound but challenging era. Sustainable developments' problem-solving networks will thus become important institutions for generations ahead and could help actualize the regional agenda 2063– The Africa we want.

## Author Contributions

SI and NU conceived, coordinated, and wrote the first draft of the manuscript. JO and NU participated in the study conception and overall study coordination. SI, MO, NU, and JO contributed in writing the subsequent drafts of the manuscript. MO and JO did the final review and edit of the draft manuscript. All authors read and approved the final draft manuscript before publication. All authors contributed to the article and approved the submitted version.

## Conflict of Interest

The authors declare that the research was conducted in the absence of any commercial or financial relationships that could be construed as a potential conflict of interest.

## References

[1] United Nations SDG Progress Reports 2019: are We on Track to Achieve the Global Goals? (2009). Available online at: https://unstats.un.org/sdgs/report/2019/# (accessed January 11, 2020).

[2] World Health Organization Leave no One Behind: Strengthening Health Systems for UHC and the SDGs in Africa. (2017). Available online at: https://www.afro.who.int/sites/default/files/2017-12/UHC%20framework_eng_2017-11-27_small.pdf (accessed January 16, 2019).

[3] Travis P Bennett S Haines A Pang T Bhutta Z Hyder AA. Overcoming health-systems constraints to achieve the millennium development goals. Lancet. (2004) 364:900–6. 10.1016/S0140-6736(04)16987-0 15351199

[4] Sachs JD From millennium development goals to sustainable development goals. Lancet. (2012) 379:2206–11. 10.1016/S0140-6736(12)60685-0 22682467

[5] Adam T Hsu J Savigny DD Lavis JN Rottingen J Bennett S. Evaluating health systems strengthening interventions in low-income and middle-income countries: are we asking the right questions? Health Policy Plann. (2012) 4:9–19. 10.1093/heapol/czs086 23014156

[6] Swanson RC Cattaneo A Bradley E Chunharas S Atun R Abbas KM. Rethinking health systems strengthening: key systems thinking tools and strategies for transformational change. Health Policy Plann. (2012) 27:54–61. 10.1093/heapol/czs090 23014154 PMC3529625

[7] Best A Holmes BJ Systems thinking, knowledge and action: towards better models and methods. J Res Debate Pract. (2010) 2:145–59. 10.1332/174426410X502284

[8] United Nations Development Program Africa Human Development Report 2012: Towards a Food Secured Future. UNDP Africa Reports. (2012). https://econpapers.repec.org/paper/agsundpar/267636.htm (accessed January 18, 2019).

[9] Castelli F. Drivers of migration: why do people move? J Travel Med. (2018) 1:1–7. 10.1093/jtm/tay040 30053084

[10] National Bureau of Economic Research The Economic Decline in Africa. (2018). Available online at: https://www.nber.org/digest/jan04/w9865.html (accessed December 28, 2019).

[11] Fosu AK Growth of African economies: productivity, policy syndromes and the importance of institutions. J Afr Econ. (2012) 22:523–51. 10.1093/jae/ejs034

[12] Dalyop GT Political instability and economic growth in Africa. Int J Econ Policy Stud. (2018) 1:217–57. 10.1007/s42495-018-0008-1

[13] Wilcox BA Colwell RR Emerging and reemerging infectious diseases: biocomplexity as an interdisciplinary paradigm. EcoHealth. (2005) 2:244–57. 10.1007/s10393-005-8961-3

[14] Zell R. Global climate change and the emergence/re-emergence of infectious disease. Int J Med Microbiol. (2004) 293:16–26. 10.1016/S1433-1128(04)80005-6 15146981

[15] Fisman D Khoo E Tuite A. Early epidemic dynamics of the West African 2014 ebola outbreak: estimates derived with a simple two-parameter model. PLoS Curr. (2014) 6. 10.1371/currents.outbreaks.89c0d3783f36958d96ebbae97348d571 25642358 PMC4169344

[16] Roberts L. Nigeria hit by unprecedented Lassa fever outbreak. Science. (2018) 359:1201–2. 10.1126/science.359.6381.1201 29590055

[17] Haider N Yavlinsky A Simons D Osman A Ntoumi F Zumla A. Passengers' destinations from China: low risk of novel coronavirus (2019-nCoV) transmission into Africa and South America. Epidemiol Infect. (2020) 148:E41. 10.1017/S0950268820000424 32100667 PMC7058650

[18] United Nations Sustainable Development Goals: Ensure Healthy Lives and Promote Well-Being for all at all Ages. (2019). Available online at: https://unstats.un.org/sdgs/report/2019/goal-03/ (accessed March 17, 2020).

[19] United Nations The Millennium Development Goals Report. (2015). Available online at: http://www.un.org/millenniumgoals/2015_MDG_Report/pdf/MDG%202015%20rev%20July%201.pdf (accessed January 15, 2020).

[20] Dovlo D Karamagi HC Ousman K Ekekemonono M Recent developments and the future of health planning in African countries. An Inst Hig Med Trop. (2017) 16 (Suppl. 1):S43–5.

[21] Murray CJL Frenk J. A framework for assessing the performance of health systems. Bull World Health Organ. (2000) 78:717–31. Available online at: https://www.who.int/bulletin/archives/78(6)717.pdf?ua=1 10916909 PMC2560787

[22] Shakarishvili G Atun R Berman P Hsiao W Burgess C Lansang MA Converging health systems frameworks: towards a concept-to-actions roadmap for health systems strengthening in low- and middle-income countries. Glob Health Govern. (2010) 3:1–17.

[23] Atun R Jongh T Secci F Ohiri K Adeyi O. Integration of targeted health interventions into health systems: a conceptual framework for analysis. Health Policy Plan. (2018) 25:104–11. 10.1093/heapol/czp055 19917651

[24] Dooris M. Expert voices for change: bridging the silos–towards healthy and sustainable settings for the 21st century. Health Place. (2013) 20:39–50. 10.1016/j.healthplace.2012.11.009 23376729

[25] Orem NJ Karamagi H Omar S Tumusiime P WHO Africa's Third Forum on Health Systems Strengthening for UHC and the SDGs. (2018). Available online at: http://www.internationalhealthpolicies.org/who-africas-third-forum-on-health-systems-strengthening-for-uhc-and-the-sdgs/ (accessed February 8, 2019).

[26] Fukuda-Parr S Greenstein J Stewart D How should MDG success and failure be judged: faster progress or achieving the targets? World Dev. (2013) 41:19–30. 10.1016/j.worlddev.2012.06.014

[27] Dovlo D Karamagi H. Life-saving hospitals-a role in UHC for Africa. Building health dreams. World Hosp Health Serv. (2016) 52:12–6. 30707807

[28] Brende B Hoie B. Towards evidence-based, quantitative sustainable development goals for 2030. Lancet. (2015) 385:206–8. 10.1016/S0140-6736(14)61654-8 25242038

[29] Uzochukwu B Onwujekwe O Mbachu C Okwuosa C Etiaba E Nystrom ME The challenge of bridging the gap between researchers and policy makers: experiences of a health policy research group in engaging policy makers to support evidence informed policy making in Nigeria. Global Health. (2016) 12:67 10.1186/s12992-016-0209-1 27809862 PMC5095957

